# The structure of the hematopoietic system can explain chronic myeloid leukemia progression

**DOI:** 10.1038/s41598-023-32400-2

**Published:** 2023-04-03

**Authors:** Mario Pérez-Jiménez, Imre Derényi, Gergely J. Szöllősi

**Affiliations:** 1 Department of Biological Physics, Eötvös Loránd University, Budapest , Hungary; 2 Department of Biological Physics, ELTE-MTA ‘Lendület’ Biophysics Research Group, Budapest , Hungary; 3 Department of Biological Physics, ELTE-MTA ‘Lendület’ Evolutionary Genomics Research Group, Budapest , Hungary

**Keywords:** Computational biophysics, Cancer models, Chronic myeloid leukaemia, Cancer stem cells

## Abstract

Almost all cancer types share the hallmarks of cancer and a similar tumor formation: fueled by stochastic mutations in somatic cells. In case of chronic myeloid leukemia (CML), this evolutionary process can be tracked from an asymptomatic long-lasting chronic phase to a final rapidly evolving blast phase. Somatic evolution in CML occurs in the context of healthy blood production, a hierarchical process of cell division; initiated by stem cells that self-renew and differentiate to produce mature blood cells. Here we introduce a general model of hierarchical cell division explaining the particular progression of CML as resulting from the structure of the hematopoietic system. Driver mutations confer a growth advantage to the cells carrying them, for instance, the *BCR::ABL1* gene, which also acts as a marker for CML. We investigated the relation of the *BCR::ABL1* mutation strength to the hematopoietic stem cell division rate by employing computer simulations and fitting the model parameters to the reported median duration for the chronic and accelerated phases. Our results demonstrate that driver mutations (additional to the *BCR::ABL1* mutation) are necessary to explain CML progression if stem cells divide sufficiently slowly. We observed that the number of mutations accumulated by cells at the more differentiated levels of the hierarchy is not affected by driver mutations present in the stem cells. Our results shed light on somatic evolution in a hierarchical tissue and show that the clinical hallmarks of CML progression result from the structural characteristics of blood production.

## Introduction

Although cancer results from stochastic mutations in somatic cells, its progression and outcome are similar across individuals^1,2^. Chronic myeloid leukemia (CML) is one of the most comprehensively studied human cancers due to its particular progression^3^. Patients are routinely diagnosed with CML in an asymptomatic chronic phase, as opposed to most cancers, in which uncontrolled cell growth is already present at diagnosis^4^. The natural course of the disease begins with an indolent chronic phase. After a few years CML patients without treatment will progress to a rapidly advancing blast phase, which is usually fatal in months^3^. In certain patients, an intermediate phase between the chronic and blast phase, called the accelerated phase, is also observed. With the development of highly efficient tyrosine kinase inhibitors (TKI) therapy, we rarely observe the natural course of the disease. Today patients rarely progress to the blast phase and the ten-year overall survival rate for CML is 80–90%^5^.

The genetic background of CML is also comparatively well understood. The fusion gene *BCR::ABL1* (resulting from a translocation between chromosomes 9 and 22) acts as a marker for mutant cells responsible for CML and is capable of inducing the disease in mice^4^. The *BCR::ABL1* fusion gene was the first mutation associated with a specific cancer type^4^.

While tumor initiation is known to be the result of the accumulation of mutations, it has recently become clear that incorporating the hierarchical structure of the tissues is also important to understand tumorigenesis^6^. As CML is a cancer of the blood, understanding its progression can only be understood in the context of blood production and the underlying hierarchical process of cell division and differentiation. Hematopoietic cells can be organized into levels according to their differentiation status. Hematopoietic stem cells (HSCs) are slowly dividing cells, dividing a few times per year^7,8^, from which the hierarchical developmental progression begins (at the top-most level). More differentiated cells lie at lower levels of the hierarchy. Terminally differentiated, mature cells are classified into two major cell types, the myeloid and lymphoid branches. Cells at each level of the hierarchy can either differentiate, producing two more specialized cells (in the level below), or self-renew, generating an additional cell at the same level. As a result of the differentiation process, cells gradually lose one or more developmental potentials and become committed to a single cell type^4^.

The bone marrow hosts cells with all maturation stages, from HSCs to fully mature blood cells. In the maturation process, cells lose their ability to self-renew and commit to differentiation. We consider four types of cells: HSCs, blast cells, committed cells, and mature cells. HSCs, described previously, produce unipotent immature blast cells. Blast cells generate immature cells committed to a particular cell type. Mature cells are fully developed and located at the bottom of the hierarchy.

The World Health Organization (WHO) had previously defined the chronic, accelerated, and blast phases based on the bloodstream blast percentage, among other criteria^9^: the thresholds for the onset of the chronic, accelerated, and blast phases being 3%, 10%, and 20%, respectively. In 2022 WHO updated the criteria for phase delineation in CML. Due to the tyrosine kinase inhibitor (TKI) therapy, the accelerated phase is rarely present in the patients, becoming less relevant. Reflecting the shift of clinical focus to resistance to treatment and development towards the blast phase, the accelerated phase has been omitted from the most recent classification^5^. Here, we keep the three-phase approach in order to compare our results against clinical data that uses this classification.

We define the onset of the latent phase of CML when the *BCR::ABL1* gene is acquired by one HSC, and this phase ends when the blast cells in the bloodstream reach 3%. This threshold is based on data about the blast percentage of newly diagnosed patients^10,11^.

The development of TKIs led to a series of mathematical models exploring CML treatment responses^1^, with an emphasis on the targeted activity of the treatment on stem cells^12^. Recently, mathematical modeling has also provided estimates of the number of levels and cell numbers for the hematopoietic hierarchy^13,14^, while incorporating neutral and driver mutations in a general model for a hierarchical tissue has started to offer insight into clonal dynamics^15^. The importance of the interaction between the stem cell and the cell-microenvironment for treatment efficiency provided new insights^16^, along with complex models involving immune response^17^.

Despite the abundance of mathematical models examining CML, however, the focus to date has been on simplified representations of the hierarchy that either do not explicitly model the bone marrow and bloodstream or do not consider driver mutations. In this study, we examine a model of hematopoiesis that incorporates both an as complete as possible representation of the bone marrow and bloodstream based on the available data, and the possible effect of driver mutations. We explore how incorporating the complete hierarchy of blood production can explain the characteristic progression pattern of CML and its role in shaping the accumulation of mutations over the course of the disease. In order to evaluate the contribution of driver mutations to CML progression we also consider gradual loss of differentiation as a possible alternative to previous models of CML, which assume a complete blockade of differentiation.

We use our extended modelling framework to investigate to what extent the structure and dynamics of the differentiation hierarchy determine CML progression. In particular, we ask whether different values of the dynamical parameters, such as the division rate of stem cells, lead to different avenues toward tumor progression. Aside from direct implications for understanding CML progression, answering this question is also potentially important for our understanding of the development and progression of tumors in other tissues.

## The model

### Qualitative description of CML progression

**Figure 1:**
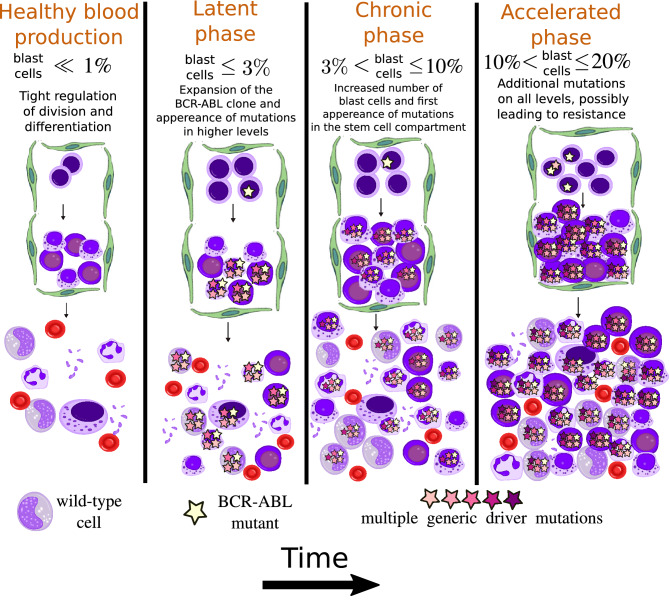
Qualitative description of CML progression. In healthy blood production with tightly regulated cell production, HSCs in the bone marrow (area surrounded by the green barrier) produce mature cells, which are released into the bloodstream (downwards). During the latent phase the *BCR::ABL1* clone grows, increasing the blast percentage in the bloodstream. In the chronic phase, the blast percentage increases, and the first generic driver mutations begin to appear. The accelerated phase is an intermediate phase, characterized by the accelerated growth of the blast percentage in the bloodstream, leading to the exponentially growing blast phase. This figure has been adapted from the original By A. Rad and M. Häggström. https://en.wikipedia.org/wiki/Haematopoiesis CC-BY-SA 3.0 license, used with permission.

In Fig. 1, we show a qualitative description of CML progression. In healthy blood production, the hematopoietic system tightly regulates the production of mature cells, corresponding to the left-most diagram. The initial growth of the *BCR::ABL1* clone produces an increased number of blast and committed cells in the bone marrow. A blast content of 3% in the bloodstream defines the end of the latent phase. This increased cellularity promotes blast cells to move through the bone marrow into the bloodstream, resulting in the chronic phase. As time continues, generic driver mutations additional to the *BCR::ABL1* mutation accumulate in the growing *BCR::ABL1* clone, increasing the self-renewal capacity and decreasing the capacity to differentiate for cells in the bone marrow. Driver mutations thus promote the increase of the number of blast cells in the bone marrow, also causing an increased movement of blast cells to the bloodstream. A group of cells that accumulate a sufficient number of driver mutations will start to grow exponentially and escape the regulated schedule of blood production.

**Figure 2:**
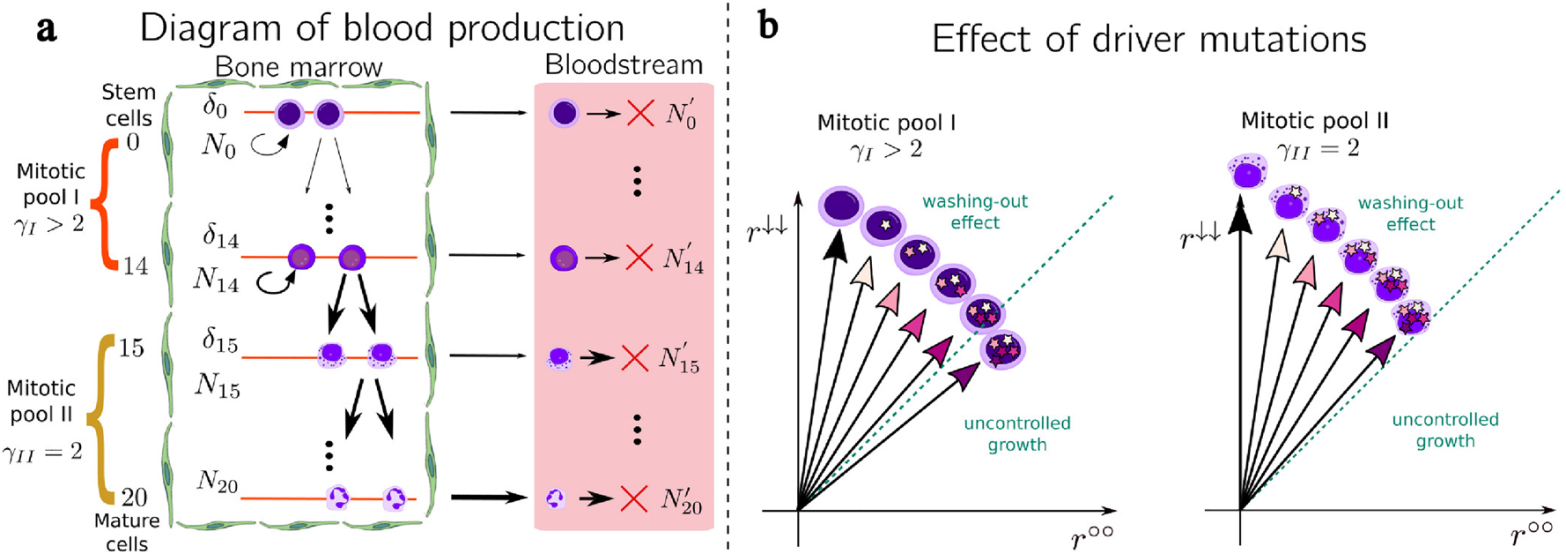
Model of blood production and the effect of driver mutations. (**a**) The bone marrow, represented by the green barrier, is comprised of two pools: mitotic pool I and mitotic pool II. Mitotic pool I contains cells with positive self-renewal rate, including stem cells. Mitotic pool II hosts cells that only produce two more differentiated cells. The horizontal arrows represent cell movement from the bone marrow into the bloodstream. The arrow width corresponds to the intensity of the cell flow. Cells in the bloodstream die with a fixed death rate per cell, represented by the red cross. (**b**) In the diagram, the horizontal and vertical components correspond to the self-renewal rate per cell and the symmetric differentiation rate per cell, respectively. Each driver mutation increments the self-renewal rate and decreases the symmetric-differentiation rate. The accumulation of driver mutations reduces the washing-out effect of the hierarchy, leading to a self-sustainable cell group. These self-sustainable cells do not require cell input from the level above. Wild-type cells in mitotic pool I require fewer driver mutations to overcome the washing-out than cells in mitotic pool II due to the former having a non-zero initial self-renewal rate. This figure has been adapted from the original By A. Rad and M. Häggström. https://en.wikipedia.org/wiki/Haematopoiesis CC-BY-SA 3.0 license, used with permission.

### Bone marrow

We base our model on previously defined models of hierarchical cell division^6,15,18,19^. In our model, cells belong to $n+1$ levels (from 0 to *n*) corresponding to their differentiation state. The top level of the hierarchy consists of HSCs, lower levels represent progressively more differentiated cells, and the bottommost level comprises mature cells. Level *k* contains $N_k$ cells. Cells at level $k<n$ can differentiate during cell division into a more differentiated level (below in the hierarchy). The number of cells produced by symmetric differentiation, per unit of time, by the $N_k$ cells at level *k* is denoted as $\delta _k$ and referred to as the symmetric-differentiation rate of the level. At the bottommost level, comprised of mature cells, cells move to the bloodstream at a rate $\delta _{n-1}$, the same rate at which the level above produces them. The cellular events occurring in the bone marrow are self-renewal, symmetric-differentiation, and mobilization. In a self-renewal event, a cell in the bone marrow divides to produce two daughter cells within the same level. In a symmetric-differentiation event, a parent cell at level *k* divides and differentiates into two more specialized cells belonging to level $k+1$. Finally, in a mobilization event, one cell from level *k* moves into the bloodstream. The amplification factor $\gamma _k$ is defined as the ratio between the symmetric-differentiation rates of two consecutive levels $\gamma _k = \frac{\delta _k}{\delta _{k-1}}$. The minimum amplification factor is 2 as detailed in the “Methods” section, cells within a level with this minimum value can only perform symmetric-differentiation. The number of HSCs has been estimated between 400 and 22, 000, we decided to implement our simulations with 10, 000 HSCs. The number of levels in the blood production hierarchy is predicted between 17 and 31 levels. The details and references for the number of HSCs and the number of levels in the hierarchy are included in the “Methods” section. For our simulations, we arbitrarily selected 21 levels for the hematopoietic hierarchy. Blood production is heterogeneous because the self-renewal and symmetric-differentiation rates change drastically for different cell types. The cell types in the bone marrow can be separated into a mitotic and a post-mitotic compartment. Cell types in the mitotic compartment have heterogeneous cell rates^4^. In our model, we divide the mitotic compartment into two sub-compartments. In mitotic pool I, cells can either self-renew or differentiate symmetrically, while cells in mitotic pool II can only differentiate symmetrically. Cells in mitotic pools I and II thus have amplification factors $\gamma _\text {I}>2$ and $\gamma _\text {II}=2$, respectively. Cells in mitotic pool I correspond to the blast cells, while committed cells belong to mitotic pool II.

In healthy adults, the median myeloblast percentage in the bone marrow is 1.4, with a range between 0 and 3^4^. We choose the boundary between mitotic pools I and II at level 15 to ensure that the number of blast cells in the bone marrow is consistent with the mentioned range. In the hierarchy, mitotic pool I comprises levels 0 to 14, and mitotic pool II contains levels 15 to 20.

### Bloodstream

Most mature blood cells perform very specialized tasks and only live for a few hours or days in the bloodstream (except for memory cells)^4^. We assume that blast and committed cells lack the ability to self-renew and differentiate symmetrically in the bloodstream. We assume that less differentiated cells will be quiescent in the bloodstream, thus having a lower death rate per cell than their counterparts in the bone marrow. Details on the definition of the death rate per cell in the bloodstream are in the “Methods” section.

### Bone marrow and bloodstream interaction

The bone marrow hosts not only blood cells but also fat cells and other skeletal elements^4^. Cellularity is the percentage of blood cells with respect to all cell types in the bone marrow and varies among different bones with age. Bone marrow cellularity is low at CML diagnosis^20^. We assume that increased cellularity leads to enhanced cell movement from the bone marrow to the bloodstream.

In Fig. 2, the horizontal arrows symbolize the movement of cells from the bone marrow to the bloodstream. We expect that differentiated cells are more prone to move from the bone marrow to the bloodstream compared with their precursors. Thus, cells in mitotic pool II should have a higher mobilization rate than cells in mitotic pool I. Mobilization intensity should also depend on bone marrow cellularity. In healthy blood production, there are primarily mature cells in the bloodstream. However, few immature cells can enter or leave the bone marrow as part of their circulation, for instance, there are four orders of magnitude more CFU-GEMM cells (a specific multi-potent blast cell) in the bone marrow than in the bloodstream^3^. Consequently, immature cells are hard to detect in the bloodstream.

### Driver mutations

A study of 39 patients in the blast phase of CML found between 0 and 4 putative driver mutations in addition to the *BCR::ABL1* fusion gene^21^. In our model, the number of driver mutations can vary depending on stochastic effects.

We consider two types of driver mutations, the specific *BCR::ABL1* driver mutation, and generic driver mutations. Generic driver mutations arise during cell division at a rate μ per cell division. A cell with a driver mutation has an increased capacity for self-renewal compared to wild-type cells. Figure 2b shows a graphical representation of the rates for mutant cells. The vertical and horizontal directions represent the symmetric-differentiation and self-renewal rates, respectively. The identity line separates two different dynamics in the hierarchy. Above the identity line, cells can be washed out of the tissue by symmetric-differentiation. On the other hand, cells below the identity line have a higher self-renewal rate than the symmetric-differentiation rate and, therefore, their probability of being washed out is very low and they can multiply exponentially.

The difference between the self-renewal and symmetric-differentiation rates is smaller for the cells in mitotic pool I compared with cells in mitotic pool II, due to the former having a positive self-renewal rate. Thus, fewer driver mutations are enough to eliminate the washing-out effect from the hierarchy in mitotic pool I than in mitotic pool II. Therefore, the number of mutations necessary to move a cell from the washing-out to the self-sustainable regions, called the critical mutation number, is smaller for cells in mitotic pool I compared with cells in mitotic pool II.

## Results

### Simulated CML progression

**Figure 3:**
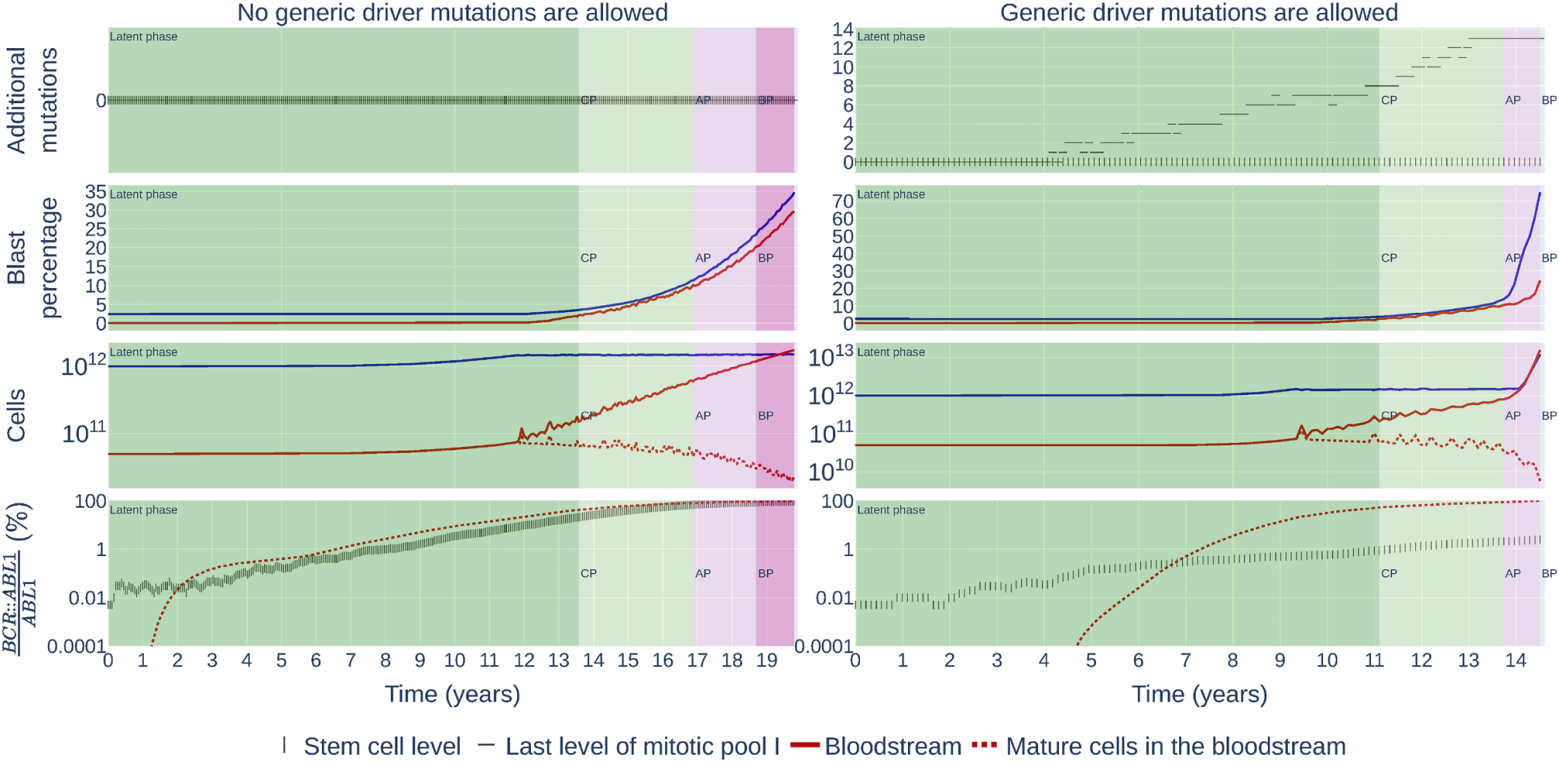
Individual instances of a computer simulation for two different progression modes. The left row corresponds to a simulation in which the mutant cells can only acquire the *BCR::ABL1* gene and no generic mutations. The simulation parameters are [mutation rate = 0, HSC divisions per year = 6.31, *BCR::ABL1* mutation strength = 0.23]. The right row depicts a simulation with additional driver mutations allowed. The simulation parameters are [mutation rate = 9 × 10^−4^, HSC divisions per year = 1.18, *BCR::ABL1* mutation strength = 0.67]. Tumor load is characterized by the *BCR::ABL1*/*ABL1* transcript level.

We qualitatively describe the events responsible for CML progression in Fig. 1. An accurate model for CML must reproduce this characteristic progression in most individual realizations. In Fig. 3, we show the course of two possible scenarios of CML progression arising from our model.

Real-time quantitative polymerase chain reaction can determine the *BCR::ABL1* transcript level ($\frac{\textit{BCR::ABL1}}{\textit{ABL1}}$) in the bloodstream. Although the level can exceed 100% (due to many factors, including upregulation of the *BCR::ABL1* in leukemic cells^1^ or loss of heterozygosity) it can be used to estimate the fraction of *BCR::ABL1* positive cells in the bloodstream. Data on the transcript level in CML patients prior to therapy exhibit a wide range of values from 50% to above 100% with an average close to 100%^1^.

The left column of Fig. 3 shows a simulation where only the *BCR::ABL1* cell clone and the healthy cells are present, while the right column shows the case where additional driver mutations are present.

In the first row, we depict the maximum number of additional mutations in a cell at two decisive levels in the hierarchy: the HSCs level and the last level of mitotic pool I. In the right column (where additional mutations occur), the maximum number of additional mutations in the last level of mitotic pool I increase rapidly up to 8 at the beginning of the chronic phase reaching 13 by the end of the chronic phase. At the stem cell level, however, no additional mutations arise.

In the second row, we show the blast percentage in the bone marrow and the bloodstream. For both progression modes, the growth of the blast percentage accelerates with time, but there is a noticeable difference at the beginning of the accelerated phase. Due to the accumulation of the additional driver mutations, the blast percentage increases more sharply, thus, shortening the duration of the accelerated and blast phases.

In the third row, both simulation modes show an increase in the total number of cells and a decrease in mature cells in the bloodstream. In the right column, we can also appreciate a sharp rise in cell numbers due to the increased cellularity produced by the self-sustainable cell group with driver mutations.

In the fourth row, we show the *BCR::ABL1*/*ABL1* transcript levels for both modes of CML progression. These levels exhibit a sharp increase after a few years from the introduction of the HSC carrying the *BCR::ABL1* gene. The leukemic cells take over and displace the healthy cells in the bone marrow over the course of the disease. An interesting observation is that if additional driver mutations are present (implying that HSCs are slower than in the left column, as explained later), then the HSC compartment still contains mainly healthy cells.

### Computer simulations predict the duration of the latent phase

A detailed explanation of the exploration of the parameter space is included in the “Methods” section, in this section we include only the final procedures. We designed a selection process to infer the parameter combinations that reproduce the characteristic CML progression. This process consists of two stages. In the first stage, we select the parameter combinations in which at least 90 out of 100 computer simulations produce a blast percentage in the bloodstream that reaches 30% within 20 years, thus, ensuring that all the CML progression phases are present. In the second stage, we select the parameter combinations in which the duration of the chronic and accelerated phases are between the desired ranges.

**Figure 4:**
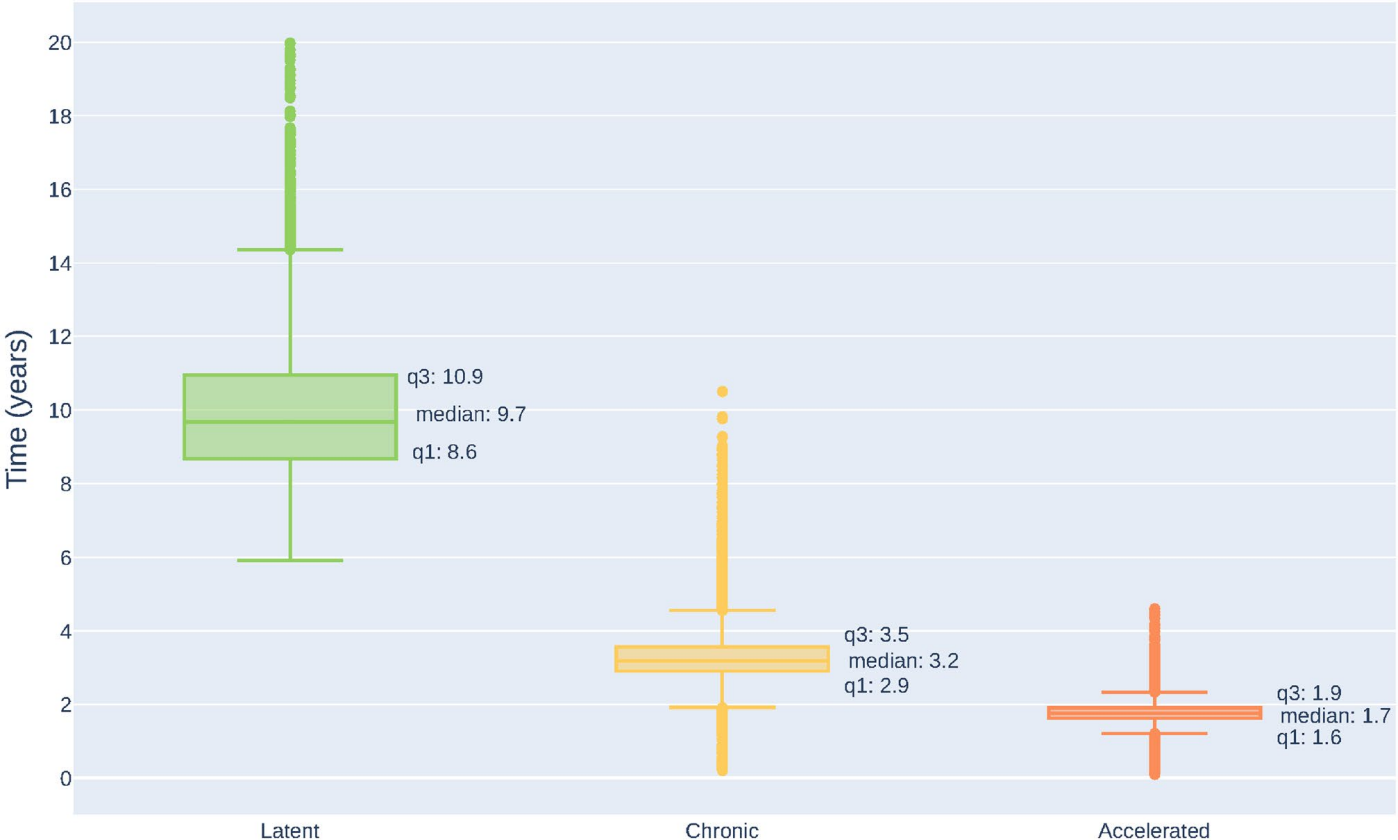
Duration of different phases of CML progression. For each parameter combination of the model, we simulated 100 instances. We selected the parameter combinations having a median duration over the 100 cases within the previously reported ranges. For these parameter combinations, we plot the distribution of the durations. All the distributions have a long tail towards long durations due to the stochastic nature of the model.

In Fig. 4 we show the phase duration histograms of the simulation instances produced with the parameter combinations selected previously. Studies based on radiobiological data report that CML incidence peaks within ten years after radiation exposure^22^. However, the data are consistent with some excess risk up to thirty years after exposure^22^. In the same report, they surveyed several models, which all predicted a latent duration distribution with an average value smaller than ten years, and with a low standard deviation, in contrast to the data.

Our model can predict the median latent phase duration using the inferred parameters as described above. Our results produce a latent phase median duration of 9.9 years, consistent with radiobiological reports. Additionally, 43% of the computer simulations have a latency longer than ten years, agreeing with the long latency period implied by previous reports. In previous models of CML, the latent phase ends when the total number of mature leukemic cells reaches a threshold. In our model, however, the blast percentage in the bloodstream is the decisive criterion for detection.

### Predictions for the relationship between the physiological parameters of CML

We noticed that the parameters related to the mobilization dynamics do not correlate with other parameters of the model. The results from our computer simulations show that the mentioned parameters have a weak effect on the duration of the CML phases. On the other hand, the three evolutionary parameters: *BCR::ABL1* mutation strength, stem cell divisions per year, and mutation rate do correlate. These parameters are also crucial for the dynamics of CML progression.

**Figure 5:**
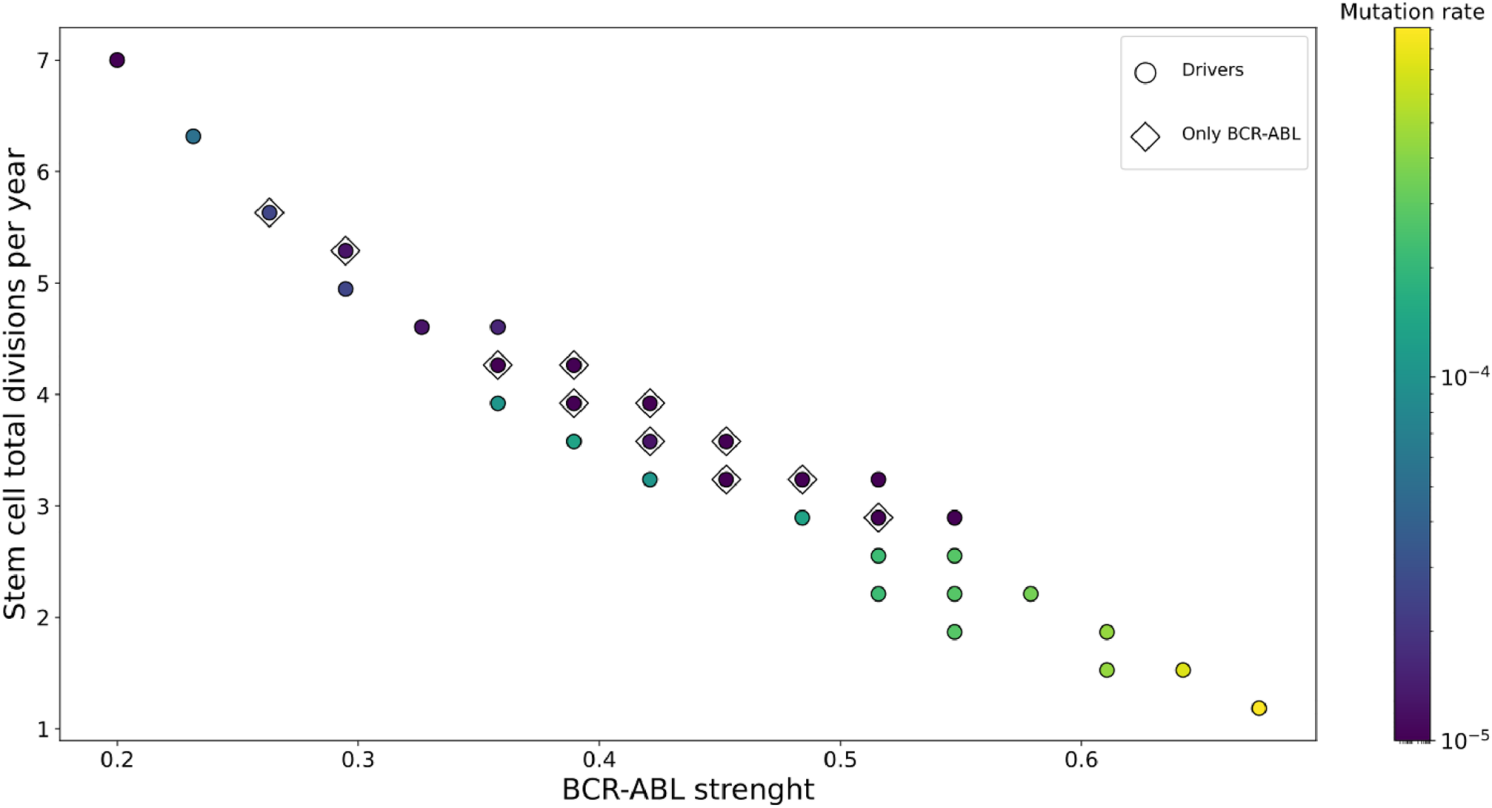
Relations between the physiological parameters of CML. The figure shows the filtered evolutionary parameter combinations for the computer simulations. The *BCR::ABL1* mutation strength negatively correlates with the stem cell divisions per year. Slow-dividing HSCs need a high-strength *BCR::ABL1* mutation to present a characteristic chronic phase and accelerated phase median duration. Diamond markers correspond to a model realization with only the *BCR::ABL1* clone allowed, which can describe CML in a smaller range of the parameters compared with those, where additional driver mutations can also occur.

In Fig. 5, we show the parameter relations revealed during the parameter fitting. These results indicate that the HSC divisions per year negatively correlate with the *BCR::ABL1* mutation strength. To test the impact of cells accumulating additional generic driver mutations on the dynamics of the simulations, we also simulated a model with only the *BCR::ABL1* clone present. For fast-dividing HSCs, the *BCR::ABL1* clone is sufficient to reproduce CML progression. However, in the case of slow-dividing HSCs, i.e. slower than two divisions per year, additional driver mutations are necessary for a proper description of CML progression.

### Number of mutations carried by cells in the hierarchy at different progression stages

**Figure 6:**
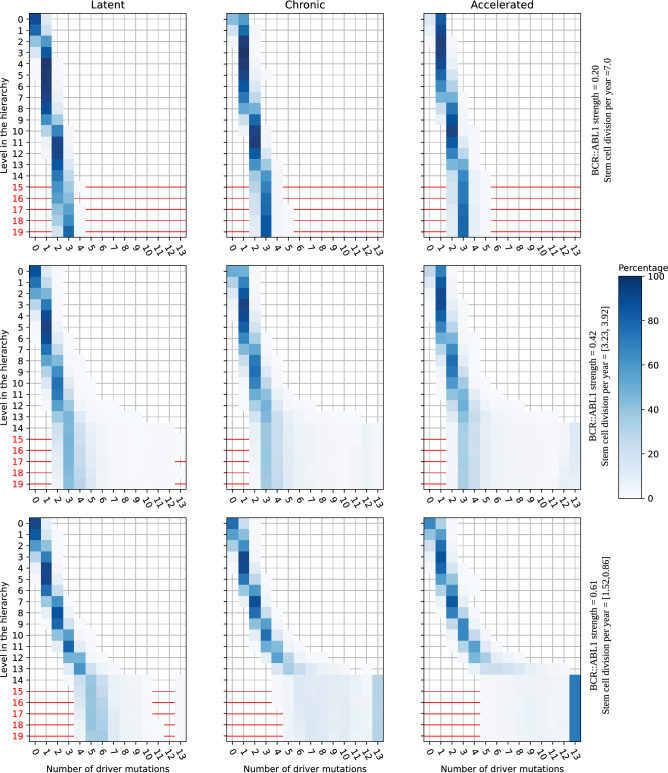
Distributions of the maximum number of generic driver mutations carried by a cell at each level of the hierarchy at the end of the three progression phases. Each row shows the mutational distribution of the hierarchy at the end of the latent, chronic, and accelerated phases for a specific value of the *BCR::ABL1* mutation strength. For each individual panel, every horizontal line displays a histogram of the maximum number of additional generic driver mutations in a cell at a given level. The color of each square with center coordinates (*x*, *y*) indicates the percentage of simulations in which *x* is the maximum number of driver mutations carried by a cell at level *y* in the hierarchy. To distinguish between mitotic pools I and II, we show the level labels of the latter in red. Cells at every level of the hierarchy develop more driver mutations with time.

Mutations carried by cells at different levels of the hierarchy shape CML progression. In Fig. 6 we show normalized histograms of the maximum number of driver mutations carried by a cell at each level of the hierarchy. We show histograms at the end of the latent, chronic, and accelerated phases. For each row, we combine all the simulations shown in Fig. 5 with a specific value of the *BCR::ABL1* strength. The color of each square with center coordinates (*x*, *y*) indicates the percentage of simulations in which *x* is the maximum number of driver mutations carried by a cell at level *y* in the hierarchy. To distinguish between mitotic pools I and II, we show the level labels of the latter in red.

Each level in mitotic pool II has $\gamma _k=2$, and the number of cells doubles that of the level above. Thus, each level in mitotic pool II has the same cell division rates. Hence, all these levels have similar mutational dynamics as shown in Fig. 6. On the other hand, levels in mitotic pool I have different dynamics. The number of driver mutations present in cells increases towards lower levels.

The bottom of Fig. 6 confirms that additional driver mutations are necessary to reproduce CML progression for slowly dividing HSCs. Almost all simulations have cells in the uncontrolled growth region in the bottom-most level of mitotic pool I. On the other hand, for fast-dividing HSCs in the top row of Fig. 6, most simulations have only cells carrying fewer mutations than the critical number for each level. For a value of the *BCR::ABL1* strength in the middle range (simultaneously with a HSC division rate in the middle range), represented in the middle row, the distribution of simulations is a combination of simulations with self-sustainable cell clones and simulations driven by the HSC clone.

Although driver mutations can accumulate at every level, it is not clear if generic driver mutations obtained at the HSC level play a role in CML progression. To answer this question we modified the model to prevent HSCs from acquiring driver mutations, but allowing other cell types acquiring generic driver mutations. We applied the Kolmogorov-Smirnov test to the duration distributions of the chronic and accelerated phases between the original and the modified model. The test indicates that both models have the same underlying distribution for the two progression phases. From this observation, we can conclude that cells at the bottom levels of mitotic pool I can accumulate additional generic driver mutations independently from the mutational profile of the HSC level.

## Discussion

Previous descriptions of CML progression propose a complete differentiation blockade caused by the accumulation of driver mutations^23,24^. Our results show that in addition to a reduction in differentiation capability caused by driver mutations CML progression can also be explained by the features of the hematopoietic hierarchy even in the case when only the *BCR::ABL1* clone is present. This, however, requires that the stem cell division rate be sufficiently fast (cf. Fig. 5).

In contrast to previous results^25^ our simulations realistically recover not only the median duration of the latent phase, but also the characteristic long tail observed in radiobiological data^22^. Our results for the transcript levels are also consistent with data from patients before starting treatment. Moreover, when assuming slow-dividing HSCs, which imply the accumulation of additional generic driver mutations, we also find that the fraction of *BCR::ABL1* positive HSCs is lower than the fraction of *BCR::ABL1* positive mature cells. This result is consistent with previous clinical observations^18,26^.

Modelling the complete differentiation hierarchy demonstrates the existence of a trade-off between the *BCR::ABL1* mutation strength and the number of HSC divisions per year. This relation is crucial for reproducing the observed CML progression, as an increase in the *BCR::ABL1* mutation strength (accelerating progression) compensates for a decrease in the division rate of HSCs (which, in contrast, slows down progression).

Our results (cf. Fig 6) show that additional driver mutations are typically accumulated by blast and committed cells (i.e., progenitor cells descending from HSCs), rather than the HSCs they descend from. We also find that by the end of the chronic and accelerated phases, HSCs have also acquired some additional driver mutations in a significant fraction of the simulations. If the mutation rate for the resistance mutations is similar to the driver mutation rate, our model will become consistent with previous reports about resistance emergence^1^. A possible avenue for future work is implementing TKI treatment and resistance emergence at different points of CML progression in the model.

Our results demonstrate the importance of the hierarchical structure of cell differentiation in tissues to understand tumor progression. Different values for the biological parameters of blood production revealed different progression scenarios. We believe that our results are important in achieving a better understanding of tumor progression in hierarchically organized tissues. Although our results do not have direct bearing on clinical practice, they are expected to be relevant for future studies of the evolution of drug resistance, especially if combined with emerging high resolutions data on patterns of somatic evolution during healthy hematopoiesis^27,28^.

## Methods

### Definition of the per cell rates

The self-renewal and symmetric-differentiation rates per cell in the bone marrow are related to the differentiation rate per level in the following way. The symmetric-differentiation rate per cell is $r_k^{\downarrow \downarrow }=\frac{1}{2}\frac{\delta _k}{N_k}$, while the self-renewal rate per cell needs to be $r_k^{{\circ }{\circ }}=\frac{1}{N_k}\left( \frac{1}{2}\delta _k-\delta _{k-1}\right)$.

The total division rate per cell is thus $R_k=\left( r_k^{\circ \circ }+r_k^{\downarrow \downarrow }\right) =\frac{1}{N_k}\left( \delta _k-\delta _{k-1}\right)$.

From $r_k^{\circ \circ } \ge 0$ it follows that $\frac{1}{N_k}\left( \frac{1}{2}\delta _k-\delta _{k-1}\right) \ge 0$, resulting in $\gamma _k \ge 2$. The case $\gamma _k=2$ represents a level in which cells only differentiate symmetrically.

### Detailed description of hematopoiesis

During leukocyte production, approximately 10^10^ myeloblasts expand and produce 1.5 × 10^11^ myelocytes in 4 cell divisions^14^. Based on this fact, previous models predict between 17 and 31 levels in the hierarchy of hematopoiesis and that the number of cells roughly doubles at consecutive levels^14^. Allometric scaling of the hematopoietic system in a large group of mammals predicts 400 HSCs actively dividing^29^. Cell labeling with division-sensitive markers and competitive transplantation studies estimate the number of HSCs in-vivo, predicting between 11, 000 and 22, 000, and the number of cell divisions between 0.4 and 2 per year^7^. Single-cell data predicted the number of HSC divisions per year between 0.6 and 6^8^. Previous models of blood production conclude that the exact number of HSCs minimally affects the dynamics of blood production^7,29^.

The median number of mature cells in the bone marrow of a healthy adult was estimated using radioisotopes between 5.6 × 10^9^ and 11.1 × 10^9^ cells/kg of body weight. Thus, the total number of mature cells in the bone marrow has a median value between 3.9 × 10^11^ and 7.7 × 10^11^ cells^30,31^. Using the same method, the total production of mature cells by the bone marrow has a median value between 5 × 10^10^ and 10^11^ cells/day^30,31^.

Considering previous data about hematopoiesis, we set the following values for the computer simulations: 10^4^ HSCs, 5 × 10^11^ mature cells in the bone marrow, and a bone marrow production of 10^11^ cells/day. We set an uneven cell distribution in the hierarchy, with the number of cells doubling in consecutive levels of mitotic pool II (between 15 and 19). We set the ratio of cells for the levels in mitotic pool I (between 0 and 14) to conform with the previously mentioned values of HSCs and mature cells in the bone marrow. This ratio can thus be expressed as $\left( \frac{5\times 10^{11}}{2^5 10^{4}}\right) ^{1/14}=2.76$. We estimated the boundary between mitotic pools I and II (at level 14) to 2.64% blast cells in the bone marrow being consistent with the estimated range between 0 and 3%^4^. In the computer simulations, we considered the HSC divisions rate as a free parameter with values between 0.5 and 6 per year.

Clinically determined transcript levels are approximated by the ratio $\left( \frac{\text {Number of mutated cells}}{\text {Number of mutated cells} + 2\times \text {Number of healthy cells}}\right) \times 100\%$.

### Physiological characteristics of peripheral blood

In a healthy adult, the median number of circulating mature cells in the bloodstream is between 5 × 10^9^ and 10 × 10^9^ cells/l. Thus the median total number of mature cells in the bloodstream lies between 2.5 × 10^10^ and 5 × 10^10^ cells^30^. At the beginning of the simulation, the average number of mature blood cells in the bloodstream is set to 5 × 10^10^ cells. The number of cells for each type at time *t* in the bloodstream is denoted by $N'_k(t)$.

### Death rate per cell in the bloodstream

The death rate per cell in the bloodstream is defined based on the total division rate per cell in the bone marrow:

1 $$\begin{aligned} r_k^{\times } = {\left\{ \begin{array}{ll} \frac{R_k}{\alpha } &{} \text {if}\, k\,\text {in mitotic pool I}; \\ R_k &{} \text {if}\, k\,\text {in mitotic pool II}; \end{array}\right. } \end{aligned}$$

where $\alpha >1$ is a parameter modifying the death rate for blast cells in the bloodstream. Blast cells are dormant in the bloodstream, thus their death rate will be much smaller than for its counterparts in the bone marrow. The parameter $\alpha$ reduces the death rate of a blast cell in the bloodstream by a factor of $\alpha$. Results from our computer simulations indicate that this parameter does not modify the duration of the CML progression phases. Thus we set $\alpha =50$, an arbitrary value that produces satisfactory simulations. The death rate for the committed cells is equal to the differentiation rate in the bone marrow due to $r_k^{\circ \circ }=0 \implies R_k=r_k$.

### Cell mobilization dynamics

Denoting the total number of cells in the bone marrow during healthy blood production as $N=\sum _{i=0}^{20}N_i(0)$, then we can denote the bone marrow cellularity at time *t* as $C(t)=\sum _{i=0}^{20}N_i(t)/N$. We assume that the cellularity growth in the bone marrow leads to a linear increase in mobilization. When the bone marrow is reaching its full capacity, the mobilization increases suddenly. Thus, we chose the following response function for mobilization:

2 $$\begin{aligned} f(C(t)) = {\left\{ \begin{array}{ll} 0 &{} \text {if}\, C(t) < 1; \\ \kappa \exp (C(t)/K) &{} \text {if}\, C(t) \ge 1, \end{array}\right. } \end{aligned}$$

where parameter *K* represents the value of the bone marrow cellularity where the cell mobilization will increase suddenly, which we consider to be equal to 10^13^ cells, setting $K=10$. The parameter $\kappa$ was optimized during the computer simulations, to produce the best description of CML progression, resulting in a value of $\kappa =0.03$.

We define the mobilization rate per cell as

3 $$\begin{aligned} r_k^{\rightarrow } = {\left\{ \begin{array}{ll} R_k f(C(t)) &{} \text {if}\, k\,\text {in mitotic pool I}; \\ R_k f(C(t))\beta &{} \text {if}\, k\,\text {in mitotic pool II}; \end{array}\right. } \end{aligned}$$

where $\beta >0$ determines the difference in the mobilization intensity between mitotic pools I and II. In a similar manner to $\alpha$ the parameter $\beta$ has no correlation with the duration of the CML progression phases and was set to 50, a value that produces adequate simulations. The parameter $\beta$ increases the mobilization rate of committed cells, while blast cells will move into the bloodstream at a lower rate.

### The effect of driver mutations

Each new driver mutation in a cell increases its self-renewal rate $r_k^{\circ \circ }$ and decrease its symmetric-differentiation rate $r_k^{\downarrow \downarrow }$. The rates are modified as follows

4 $$\begin{aligned} \tilde{r_k}^{\circ \circ }&=r_k^{\circ \circ }+s\Delta R_k \end{aligned}$$

5 $$\begin{aligned} \tilde{r_k}^{\downarrow \downarrow }&=r_k^{\downarrow \downarrow }-s\Delta R_k, \end{aligned}$$

where the rates with a tilde represent the mutated cell rates, and the rates without a tilde are the cell rates before the mutation. Parameter $s \in [0,1]$ is the mutation strength. The mutation strength is a fraction of the minimum mutation strength necessary to produce a self-sustainable cell in the last level of mitotic pool I $\Delta = \frac{r_{14}^{\downarrow \downarrow }-r_{14}^{\circ \circ }}{2R_{14}}$. A driver mutation adds a fraction of the symmetric-differentiation rate to the self-renewal rate, preserving the total cell division rate $R_k$.

To parameterize the driver mutations, we define the *BCR::ABL1* mutation strength as $s_{\textit{BCR::ABL1}}$. In Fig. 5 the *BCR::ABL1* strength reported is the value of $s_{\textit{BCR::ABL1}}$. We expect that few driver mutations are sufficient for CML progression. Thus, we choose $m_\text {crit}=4$. We did not optimize this value in the computer simulations. All the generic mutations have the same mutation strength $\frac{1-s_{\textit{BCR::ABL1}}}{m_\text {crit}}$. This ensures that a cell with the *BCR::ABL1* mutation and four generic driver mutations is self-sustainable.

### Implementation of the computer simulations

The simulation is based on Gillespie’s algorithm. It starts with the introduction of an HSC with the *BCR::ABL1* mutation into a healthy blood production process. This single HSC will be responsible for the initiation of CML. The simulation will stop when any of the following two conditions are satisfied. The simulation has been running for more than 20 years, or the blast percentage in the bloodstream is higher than 30%.

The algorithm does not keep track of each individual cell, but only the numbers with different states for the bloodstream and bone marrow compartments. The state of a cell is characterized by the differentiation level, the presence of the *BCR::ABL1* mutation, the number of genetic driver mutations, and the respective compartment.

### Initial exploration of the parameter space

First we performed a rough exploration of the parameter space of the model with simulations of 9417 parameter combinations in a realistic range for each parameter. After analyzing the results, we selected a region in the parameter space that produced the desired dynamics on the simulation. For a refined search in this parameter region, we explored 541 parameter combinations. The final part of the parameter exploration process is described in section *Computer simulations predict the duration of the latent phase*.

## Data Availability

The computer code to simulate CML progression and reproduce the figures are available on https://github.com/marioperezj/cml_progression_model. The data generated and analysed during the current study are available in the repository https://github.com/marioperezj/cml_progression_model/tree/master/data.
